# Supplementary material for: Single Cell Transcriptomic and Chromatin Profiles Suggest Layer Vb Is the Only Layer With Shared Excitatory Cell Types in the Medial and Lateral Entorhinal Cortex
Source: Front Neural Circuits. 2022 Jan 26;15:806154. doi: 10.3389/fncir.2021.806154 (PMC8826650; doi:10.3389/fncir.2021.806154)
Supplement: Supplementary file 14 [file Data_Sheet_1.ZIP › Sup_vs_Deep_Motifs/homerResults/motif3.similar.html]

motif3

## Information for motif3

C
A
T
G
T
C
G
A
A
C
G
T
A
C
T
G
C
G
T
A
T
A
C
G
A
G
C
T
G
T
A
C
C
G
T
A
A
G
C
T
  
Reverse Opposite:  

T
C
G
A
A
C
G
T
A
C
T
G
C
T
G
A
A
T
G
C
A
C
G
T
T
G
A
C
C
G
T
A
A
G
C
T
G
T
A
C
  

|  |  |
| --- | --- |
| p-value: | 1e-248 |
| log p-value: | -5.728e+02 |
| Information Content per bp: | 1.693 |
| Number of Target Sequences with motif | 4409.0 |
| Percentage of Target Sequences with motif | 13.16% |
| Number of Background Sequences with motif | 664.2 |
| Percentage of Background Sequences with motif | 7.78% |
| Average Position of motif in Targets | 291.2 +/- 234.0bp |
| Average Position of motif in Background | 339.2 +/- 268.6bp |
| Strand Bias (log2 ratio + to - strand density) | 0.0 |
| Multiplicity (# of sites on avg that occur together) | 1.09 |
| Motif File: | file (matrix) reverse opposite |

### Similar de novo motifs found

|  |  |  |  |  |  |  |  |
| --- | --- | --- | --- | --- | --- | --- | --- |
| Rank | Match Score | Redundant Motif | P-value | log P-value | % of Targets | % of Background | Motif file |
| 1 | 0.960 | C G T A C T A G T G C A A C G T A C T G C G T A A T C G A C G T A G T C C G T A A T G C G C A T | 1e-225 | -519.846417 | 11.46% | 6.67% | motif file (matrix) |
| 2 | 0.972 | T C G A A C G T A C T G C G T A T A G C A G C T G T A C C T G A | 1e-209 | -482.894136 | 18.54% | 12.60% | motif file (matrix) |
